# Supplementary figures and images for: Static and dynamic alterations in the amplitude of low-frequency fluctuation in patients with amyotrophic lateral sclerosis
Source: PeerJ. 2020 Nov 2;8:e10052. doi: 10.7717/peerj.10052 (PMC7643554; doi:10.7717/peerj.10052)

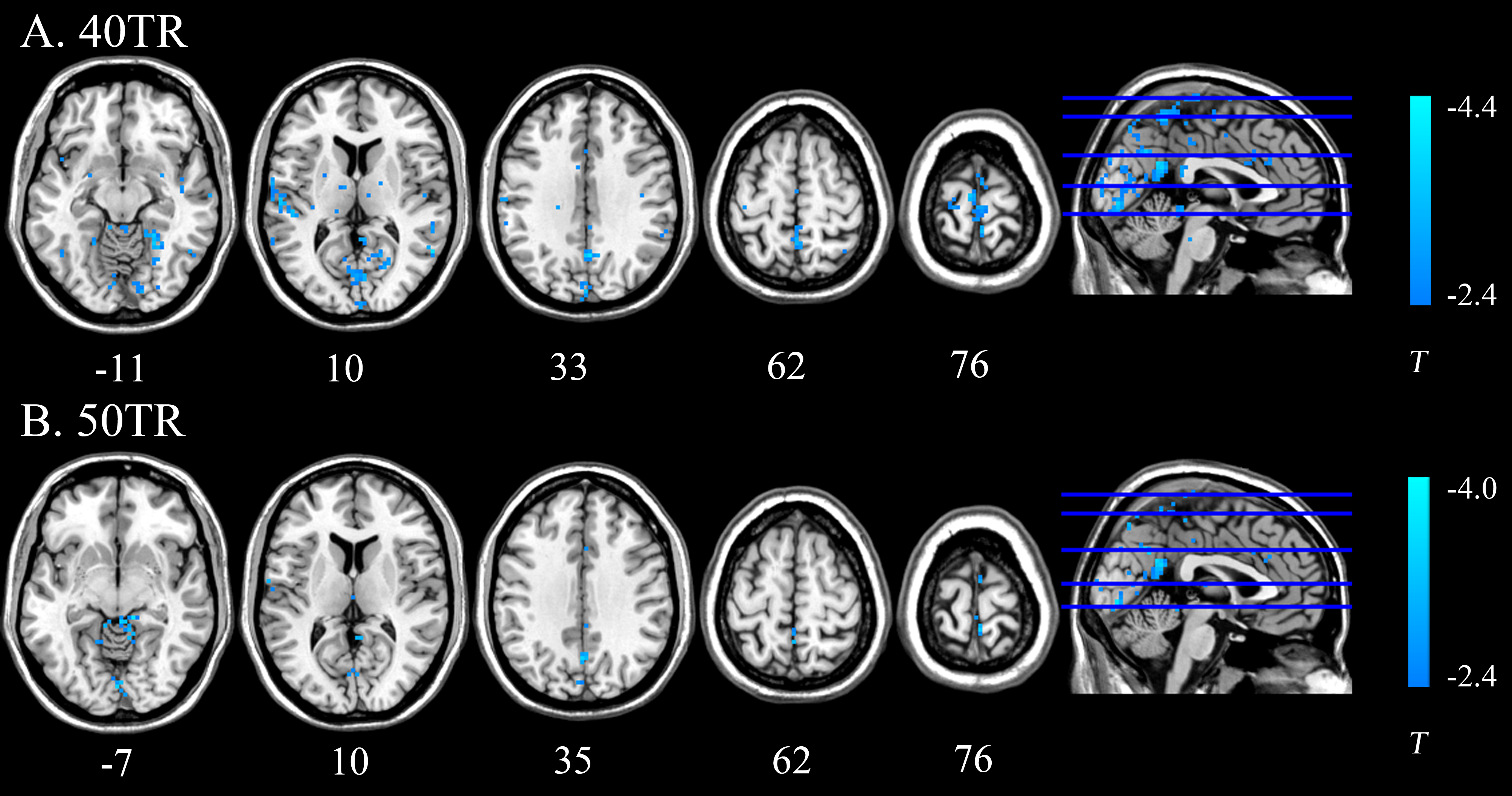

Supplement: Figure S1 — a. Results of the sliding-window length of 40 TR. b. Results of the sliding-window length of 50 TR. [file peerj-08-10052-s002.png]
